# Identification of a novel *WAS* mutation and the non-splicing effect of a second-site mutation in a Chinese pedigree with Wiskott–Aldrich syndrome

**DOI:** 10.1186/s13023-022-02589-y

**Published:** 2022-12-22

**Authors:** Xin Ji, Xuening Hou, Xin Guo, Yifeng Sun, Futian Ma, Jihong Hao

**Affiliations:** 1 Department of Clinical Laboratory, The Second Hospital of Hebei Medical University, 215#, Heping West Road, Shijiazhuang, Hebei Province, 050000 China; 2 Department of Hematology and Oncology, Children’s Hospital of Hebei Province, 133#, Jianhua South Street, Shijiazhuang, Hebei Province, 050031 China

**Keywords:** Wiskott–Aldrich syndrome, Microthrombocytopenia, Novel mutation, Splicing effect, In vitro models

## Abstract

**Background:**

Wiskott–Aldrich syndrome (WAS) is a rare X-linked immunodeficiency disorder caused by abnormal expression of the WAS protein (WASp) due to mutations in the *WAS* gene, and is generally characterized by microthrombocytopenia, eczema, recurrent infections, and high susceptibility to autoimmune complications and hematological malignancies.

**Results:**

Herein, we identified a novel *WAS* mutation (c.158 T > C) using next-generation sequencing in a Chinese pedigree with WAS. The expression of WASp in the patients and their families was detected by flow cytometry and western blot analysis. To explore the exon-splicing effect of intron mutations and the correlation between the genotype and clinical phenotype, four groups of wild-type (WT), exon mutant, intron mutant, and combined mutant recombinant plasmids were transfected into COS-7 cells in vitro. The proband showed dramatically decreased WASp expression, while the female carriers showed a slightly lower level of WASp. The expression of products in the mutant and WT recombinant plasmids was detected by real-time fluorescence quantitative polymerase chain reaction (PCR), which showed a significant reduction in the combined mutant group than in the WT, exon mutant, and intron mutant groups. The length of the expression products in the four groups showed no differences, each containing 360 base pairs. Sequence analysis confirmed that the c.158 T > C mutation appeared in the exon mutant and combined mutant groups, whereas the intron variant c.273 + 14C > T caused no other sequence changes.

**Conclusion:**

This study confirmed that the intron mutation did not affect the splicing of exons and excluded the influence of the double mutations at the transcription level on the severe clinical manifestations in the cousin. This in vitro study provided new insights into the pathogenesis of intronic mutations in WAS.

## Background

Wiskott–Aldrich syndrome (WAS: OMIM 301,000) is a rare X-linked recessive immunodeficiency disease, that is usually characterized by the triad of eczema, recurrent infections and microthrombocytopenia leading to bleeding. Patients with severe WAS are susceptible to the development of autoimmune diseases, lymphoma or other malignancies. The severity of clinical manifestation is scored according to the degree of thrombocytopenia (score, 0.5–1), eczema and immunodeficiency (score, 2–4), and the presence of autoimmunity, malignancy or even death (score 5) [1, 2]. Generally, affected males show clinical manifestations at an early age, whereas females carrying the defective *WAS* gene are asymptomatic. The incidence of WAS is less than 1 in 100,000 live births [3]. Patients who show only thrombocytopenia initially are prone to be misdiagnosed as having immune thrombocytopenia (ITP), which may result in inappropriate treatment and delayed life-saving therapy [4]. Hematopoietic stem cell transplantation (HSCT) is the gold standard treatment for patients with WAS. The earlier HSCT for WAS patients is performed, the higher the survival rate [5, 6].

The *WAS* gene, which is located on Xp11.22–p11.23, is responsible for this disease. This gene consists of 12 exons that encode Wiskott–Aldrich syndrome protein (WASp), which contains 502 amino acid and participates in the regulation of actin cytoskeleton-dependent cellular processes, such as cell signaling, migration, immune synapse formation, and cytokine release [7, 8]. The *WAS* gene contains an Ena/VASP homology 1 (EVH1/WH1) domain, a basic region (BR), a GTPase-binding domain, a proline-rich region and the verprolin, cofilin and acidic region from the N terminus to the C terminus. Missense mutations, considered the most common mutations of *WAS*, mostly occur in exons 1–4 of the WAS-interacting protein (WIP) binding domain [9]. Splice-site mutations are commonly located in introns 6–10, whereas insertions and deletions occur across the *WAS* gene [10, 11]. The classic WAS is accompanied by entire loss-of-function mutations, while partial loss-of-function mutations result in X-linked thrombocytopenia (XLT) with decreased levels of WASp and gain-of-function mutations lead to the rare X-linked neutropenia (XLN) [12, 13].

Although the genotype–phenotype connection of WAS/XLT has not been fully clarified, several studies have revealed that the clinical phenotype of typical WAS or milder XLT is potentially influenced by the effects of these mutations and WASp expression [11, 14, 15]. Milder WAS symptoms are frequently associated with missense and splice-site mutations, whereas severe WAS symptoms are associated with nonsense and frameshift mutations [10, 16]. Moreover, patients with missense mutations that allow the expression of WASp present with mildly symptomatic XLT, showing a better prognosis [17].

**Fig. 1:**
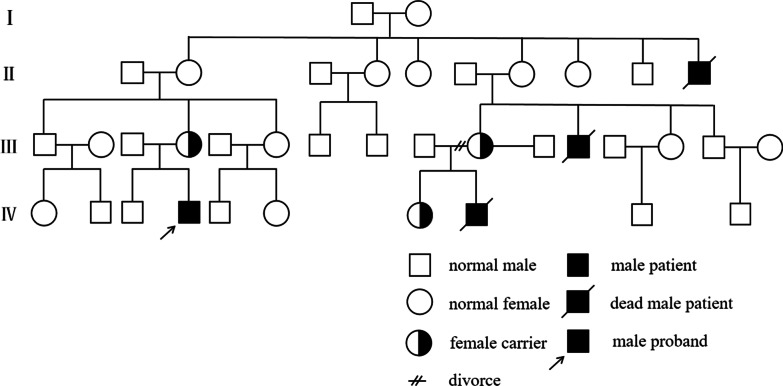
The pedigree of the WAS proband

Here, we report a novel mutation in *WAS* in a Chinese family (Fig. 1). The proband had typical features of WAS with a novel missense mutation in exon 2, and his cousin who presented with severe symptoms of thrombocytopenia, hemorrhage and recurrent infections was discovered to have a second-site mutation in intron 2. Therefore, this study explored whether the intron mutation affected exon splicing and evaluated the correlation between the second-site mutation and the aggravated phenotype in the cousin.

## Results

### WASp expression

**Fig. 2:**
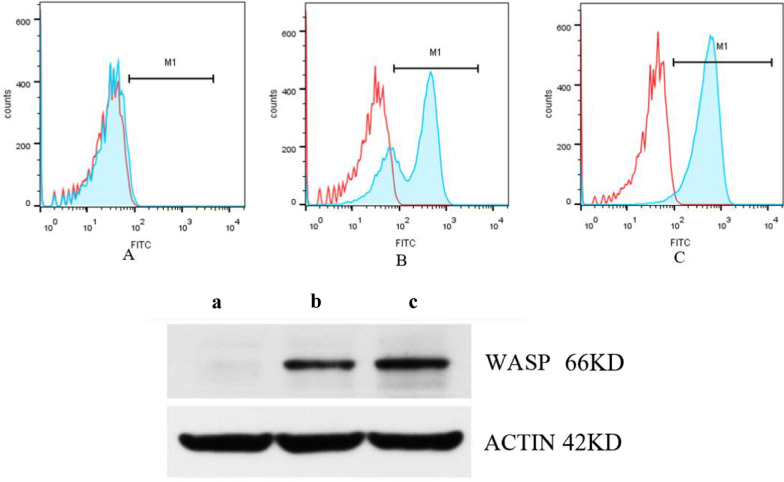
Expression of WASp in the proband and his mother using flow cytometry analysis on the above. **A** The proband (3.8%); **B** his mother (72.8%); **C** normal individual (96%); Expression of WASp in the proband and his mother performed by western blot analysis on the below. (a) the proband; (b) his mother; (c) normal control

**Fig. 3:**
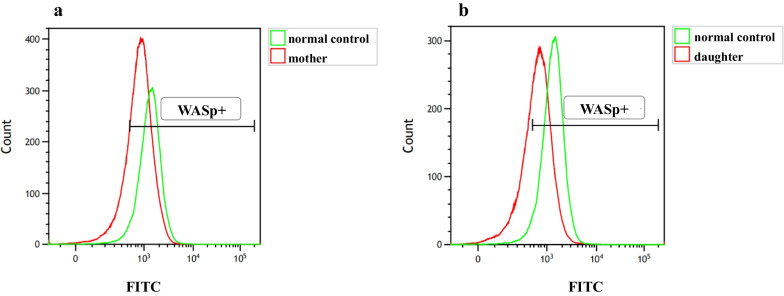
Expression of WASp in the patient 2’s mother and his younger sister using flow cytometry analysis. **a** Mother of patient 2 (77.68%); **b** the younger sister of patient 2 (64.58%) and normal individual (95.11%)

The intensity of WASp expression in peripheral blood mononuclear cells (PBMCs) isolated from the proband and his mother was 3.8% and 72.8%, respectively, as determined by flow cytometry (Fig. 2). The intensity of WASp expression in the PBMCs of the patient was significantly lower than in normal individual (96%). Western blot analysis showed that WASp expression in the proband was absent in comparison with that in his mother and normal control (Fig. 2). The intensity of WASp expression in patient 2’s mother and his sister was 77.68% and 64.58%, respectively, while that in the normal control was 95.11% (Fig. 3).

### Mutation analysis

**Fig. 4:**
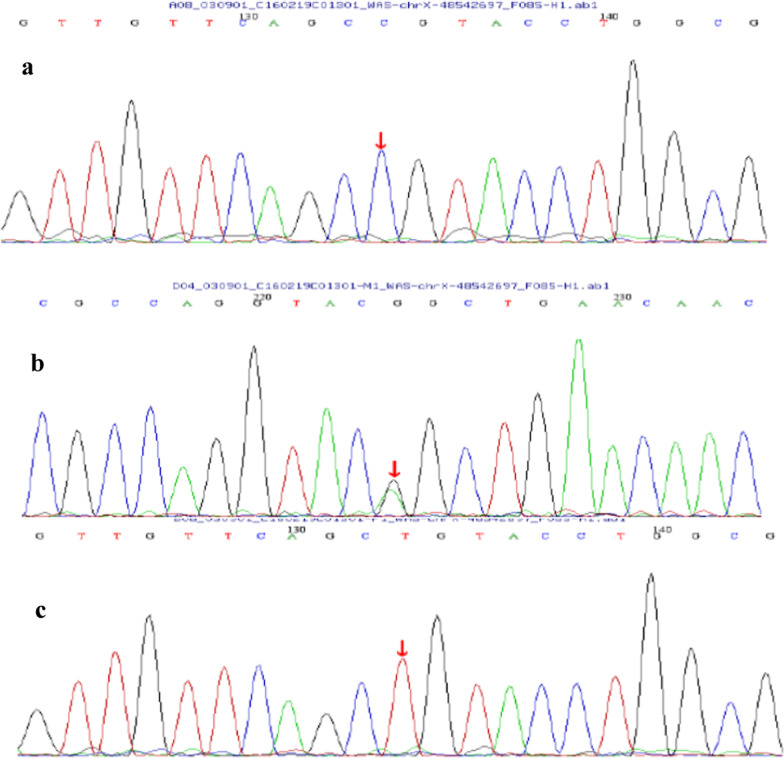
**a** Mutation analysis of *WAS* gene in the proband (hemizygous mutation of c.158 T > C in exon 2, p.L53P); **b** Sequencing analysis of *WAS* gene in the proband’s mother (heterozygous mutation of c.158 T > C); **c** Sequencing analysis of *WAS* gene in the proband’s father (wild-type with no mutation)

**Fig. 5:**
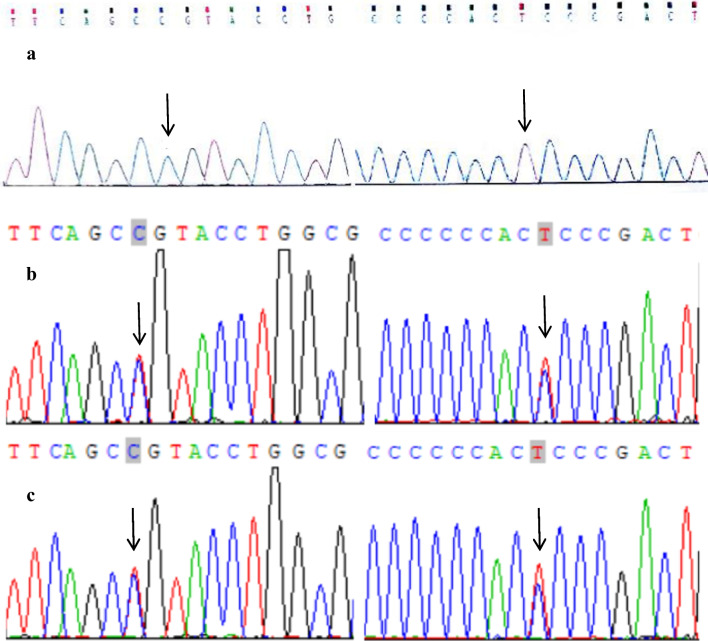
**a** Mutations in *WAS* gene of the younger cousin (c.158 T > C in exon 2 and c.273 + 14C > T in intron 2); **b** Mutations in *WAS* gene of the cousin's mother (heterozygous mutation of c.158 T > C in exon 2 and c.273 + 14C > T in intron 2); **c** Mutations in *WAS* gene of the cousin’s sister (heterozygous mutation of c.158 T > C in exon 2 and c.273 + 14C > T in intron 2)

Sequence analysis showed that the proband and his cousin had a hemizygous missense mutation (c.158 T > C) in exon 2 of the *WAS* gene, which was predicted to change leucine to proline (p.L53P) (Fig. 4a). The heterozygous mutation 158 T > C was also confirmed in the proband’s mother (Fig. 4b), whereas no mutation was detected in his father (Fig. 4c). In addition to 158 T > C, the cousin also had a second-site mutation c.273 + 14C > T (Fig. 5a); his mother showed the heterozygous mutations 158 T > C and c.273 + 14C > T (Fig. 5b), while his sister was also a WAS carrier with heterozygous mutations (Fig. 5c).

Based on the consensus *WAS* sequence (ENST00000376701), online mutation analysis using Mutation Taster and PolyPhen-2 indicated that mutation 158 T > C in exon 2 may be a pathological factor. Mutation Taster indicated that the protein features might have been affected (http://www.mutationtaster.org/cgi-bin/MutationTaster/Mutation Taster69.cgi), while PolyPhen-2 predicted probable damage with a score of 1.00 (http://genetics.bwh.harvard.edu/ggi/pph2/7e5771b52fb6b1008d4a14d80a963e961bba4690/6293018.html).

### Expression products of the recombinant mutant vectors in COS-7 cells

**Fig. 6:**
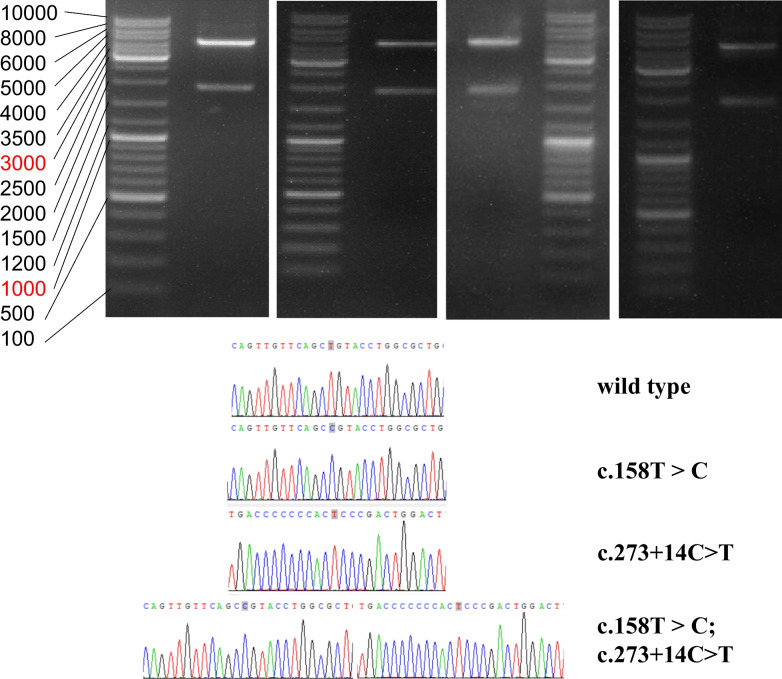
The restriction endonuclease analysis of four recombinant vectors in pET-01 on the left. The restriction enzymes are *XhoI* and *BamHI*. The expected size is 1849 bp and 4500 bp. The two bands show approximately 1800 bp and more than 4000 bp using electrophoresis. The gene sequencing map of the recombinant vectors on the right respectively represents wild-type, exon mutant, intron mutant and combined mutant group

After enzymatic digestion with *XhoI* and *BamHI*, the polymerase chain reaction (PCR)-amplified gDNA showed two bands on agarose gel electrophoresis (Fig. 6): a 1849-bp band and a more than 4000-bp band, each of which corresponded to the length of the plasmid pET-01. We extracted these two bands and amplified them under the same PCR conditions. Direct sequencing confirmed that the short bands in the four groups were errorless for the target mutations (Fig. 6).

**Fig. 7:**
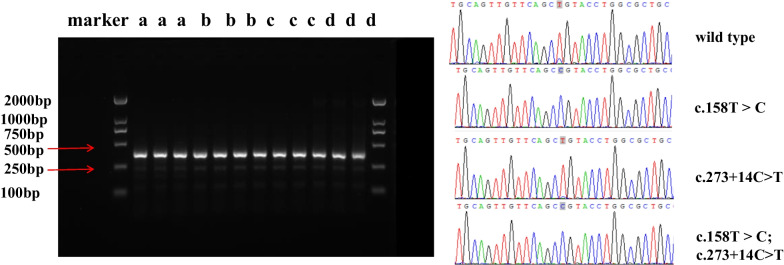
cDNA with a length of 360 bp in COS-7 cells transfected with pET-01 recombinant vector. **a** combined mutant group; **b** intron mutant group (c.273 + 14C > T); **c** exon mutant group (c.158 T > C); **d** wild-type group. Sequencing analysis of 4 cDNA fragments containing the first three exons of *WAS* gene

The empty plasmid vector served as a negative control, and the relative transcript levels of the four groups were shown in Table 1. The combined mutant group showed significantly lower transcript levels than the wild-type (WT), exon mutant and intron mutant groups. However, the length of the expression products of four recombinant mutated vectors in COS-7 cells showed no differences. Meanwhile, sequence analysis confirmed the appearance of the c.158 T > C mutation in the exon mutant group and combined mutant group, whereas the intron variant c.273 + 14C > T caused no abnormal sequences variation in this part of *WAS* (Fig. 7). No differences were detected among the four cDNA fragments.

**Table 1:** Relative expression of *WAS* gene containing the first three exons by RT-qPCR

Number	Group	Relative expression of partial *WAS* gene	The average value of relative expression
1	Empty plasmid^①^	1.000	0.957
	Empty plasmid	0.982	
	Empty plasmid	0.889	
2	Wild-type^②^	1636.805	1681.849
	Wild-type	1694.527	
	Wild-type	1714.216	
3	c.158 T > C	2568.422	2498.951
	c.158 T > C	2418.673	
	c.158 T > C	2509.759	
4	c.273 + 14C>T	2115.329	2055.928
	c.273 + 14C>T	1942.008	
	c.273 + 14C>T	2110.448	
5	Combined mutation^③^	891.444	881.948
	Combined mutation	887.334	
	Combined mutation	867.067	

①: No target gene fragment was inserted; ②: gDNA of *WAS* including exon 1, 2, 3 and intron 1 and 2 (1849 bp) as the target gene was inserted; ③: The first three exon of gDNA containing the two mutations (c.158 T > C; c.273 + 14C>T) was inserted

## Discussion

WAS is a complex X-linked primary immunodeficiency disorder along with thrombocytopenia. Persistent thrombocytopenia with small platelets is a pathogenetic sign of WAS/XLT, presenting spontaneous or posttraumatic bleeding. The degree of bleeding varies from petechiae or ecchymosis to severe hematomas and even to life-threatening intracerebral or intestinal hemorrhages [1]. The patients in our study presented with varying degrees of bleeding, including petechiae, ecchymosis, and infrequent umbilical hemorrhage. In fact, continuous microthrombocytopenia can be attributed to accelerated destruction of defective platelets in the spleen combined with the co-action of antiplatelet autoantibodies [18]. However, due to the early onset of thrombocytopenia, the intermittent low platelet count can hamper accurate diagnosis of WAS; patients with WAS/XLT, especially XLT [19], are often misdiagnosed as showing ITP, and only 7% of these patients are ultimately diagnosed [20]. The proband and his cousin in the present case were both initially misdiagnosed with ITP at an early age. Although the platelet count improved temporarily after treatment with dexamethasone sodium phosphate and immunoglobulin, the patients later showed significantly decreased platelet count and progressed to more severe clinical manifestations, such as severe immune dysregulation with susceptibility to infections, eczema.

Based on the Human Gene Mutation Database (HGMD), there are more than 450 unique mutations across all 12 exons, including nonsense, missense, insertion, deletion, rearrangement, and splice-site mutation. In our report, mutation analysis identified a novel mutation of 158 T > C in the proband and his younger male cousin, which caused a change in leucine to proline at the 53rd amino acid. Missense mutations in exons 1, 2, and 3 of the *WAS* gene are the most common mutations in patients with milder phenotypic features, such as mild XLT [15]. However, both patients had typical WAS symptoms and were clinically assigned scores of 4 and 5 respectively. This missense mutation residing in the N-terminal WIP binding site can impair the affinity of WASp for WIP, thus leading to dissociation of the complex and degradation of WASp [21]. In addition, the mutation may lead to defective PLT production by megakaryocytes in the bone marrow, thereby affecting the number and volume of platelets [22].

More studies have identified various clinical phenotypes associated with diverse mutations in the *WAS* gene, indicating a close genotype–phenotype correlation with a certain predictability for clinical outcomes. The mutations 168C > T (T45M), 290C > N/291G > N (R86C/H/L) and IVS6 + 5 g > a consistently resulted in reduced WASp and relatively mild clinical manifestations whereas 665C > T (R211X), IVS8 + 1 g > n and IVS8 + 1 + 6del gtga predominantly represented the absence of WASp expression and a severe clinical phenotype; these six hotspot mutations accounted for approximately 25% of all mutations [11, 23]. Mutations adjacent to the 158 T > C in our case such as 151G > T (Val51Phe), 156G > C (Gln52His), and 162C > A (Tyr54Tem) contribute to the classical WAS phenotype [24–26]. Furthermore, mutations 155A > C (Gln52Pro) and 167C > T (Ala56Val) lead to the XLT phenotype and patients with 166G > A (Ala56Val) experienced intermittent XLT [23, 27, 28].

The absence of WASp or truncated proteins is typically associated with severe clinical symptoms in affected individuals. The decreased expression of WASp in the proband corresponded to his clinical manifestations. Meanwhile, the WASp expression in his mother, who was a heterozygous carrier, was only slightly reduced due to the defective *WAS* gene which might represent preferential X-chromosome inactivation (XCI) of the female WAS carrier that allowed survival of partially defective WAS cells [29].

Up to 11% of patients with WAS present with somatic mosaicism due to spontaneous reversion of the original mutation to normal or the appearance of second-site compensatory mutations, which partially restore the biological function of WASp [30]. Wada et al. [31] described a second-site mutation causing the deletion of 19 nucleotides from nucleotides 1299 to 1316 in two brothers with WAS, suggesting that the original mutation (1305insG) resulted in frameshift and abrogated protein expression, while the second-site mutation revised the adverse effects of the original mutation. Notably, a c.273 + 14C > T second-site mutation was detected in the younger male cousin in this study, who showed more severe clinical manifestations than the proband and died of fungal infection at 3 years of age. To date, splice-site mutations have been mostly shown to be distributed in intron 6–11 [15, 16] and are rarely reported in intron 2. Ariga et al. firstly performed a mutation in the acceptor site of intron 2 of the *WAS* gene (IVS2-1 g > a) and confirmed that the abnormal lack of exon 3 was due to the splicing defect [32]. In addition, IVS2 + 1 g > a and IVS2 + 1 g > t both resulted in the absence of WASp; however, the splicing effect was not determined [11]. Similarly, the other two mutations (IVS2 + 2t > c and IVS2-2a > c) were associated with predictive splicing abnormalities [25, 33].

Due to the unavailability of the cousin’s blood, we used a recombinant mutant vector in COS-7 cells in vitro to explore whether the second-site mutation in intron 2 caused the cousin’s more severe clinical manifestations compared to the proband. The results showed that the relative expression of the *WAS* gene, as determined by real-time fluorescence quantitative PCR (RT-qPCR), in the combined mutation was approximately 50% lower than that in the WT group (Table 2). However, the four groups showed no differences in the length and sequence of expression products, confirming that the intron variant c.273 + 14C > T had no effect on RNA splicing in this part of the *WAS* gene. The ratio of CD4 + and CD8 + T cells, which is closely related to opportunistic infections, was 0.3, indicating cellular immune dysfunction and a higher probability of opportunistic infection. Multiple opportunistic infections were most likely an important culprit for the rapid exacerbation of clinical symptoms and death of the younger cousin. Therefore, we speculated that the cousin had already suffered severe multiorgan damage or irreversible complications when recovery mutations occurred. Although revertant mutations could have resulted in partial immune or hematopoietic function reconstruction [34], the cousin died of severe infections that could not been reversed by single second-site mutation.

**Table 2:** The laboratory examination data of the proband and his brother

Variable	Proband	The proband’s brother	Normal range
WBC (× 10^9^/L)	22.82	16.1	3.5–9.5
Hb (g/L)	113	73	120–160
PLT (× 10^9^/L)	22	44	125–350
IgG (g/L)	6.3	18.10	7.5–15
IgA (g/L)	0.02	0.76	1.0–5
IgM (g/L)	0.12	1.6	0.46–3
IgE (mg/L)	2.9	4.93	0.1–0.9
EBV-DNA (× 10^3^ IU)	2	Negative	< 1
HCMV-DNA (× 10^3^ IU)	Negative	8.027	< 0.5
total T cell (%)	52.1	85.8	20–50
CD3 + (%)	48.8	–	56–86
CD3 + CD4 + (%)	34.4	12.4	33–58
CD3 + CD8 + (%)	13.5	36.8	20–39
CD3 + CD4 + /CD3 + CD8 + (%)	2.55	0.3	0.7–2.5
CD19 + /20 + (%)	7.7	–	5–18
CD3-CD56 + (%)	34.3	7.8	10–20

– means that the data is unattained

After excluding the splicing effect of the second-site mutation at the experimental molecular level, several factors may have aggravated the clinical manifestations in the cousin. First, some WAS patients may have already suffered severe multiorgan damage or irreversible complications, such as malignancies, before the occurrence of the recovery mutations [34]. Thus, although revertant mutations can facilitate partial immune or hematopoietic function reconstruction, they are not sufficient to maintain long-term survival of patients with WAS. In this regard, the cousin died of severe infections that could not be reversed by single second-site mutation. Second, age of onset and disease progression are also factors influencing the effects of second-site mutations. A retrospective analysis of 160 patients revealed that the risk of developing a clinical score of 5 was significantly higher during the first 2 years of life [2].

Gene therapy has emerged as an effective treatment option for WAS, and successful gene therapy procedures for WAS will increase the availability of a cure for patients with WAS and possibly reduce the potential side effects associated with mismatched allogeneic transplantation [35]. Nevertheless, HSCT remains the most reliable curative treatment, and shows excellent results for patients with WAS. Thus, prompt and correct diagnosis is of vital significance for patients with WAS to improve their quality of life and increase their survival rate.

## Conclusions

In general, the results of this in vitro study supported the lack of splicing effects of the second-site mutation, thus indicating its weak function in the clinical phenotype. Genetic analysis and a review of family history are important for confirming the diagnosis of patients with WAS. In this regard, the results of effective research studies can be expected to substantially contribute to the diagnosis and treatment of WAS. More relevant studies are needed to identify the mechanism of phenotypic impact of second-site mutation in these cases.

## Methods

### Case presentation

Patient 1, a six-month-old boy, was initially admitted to our hospital in April 2014 with thrombocytopenia, lung infections, bleeding, and ecchymosis. The complete blood count showed the following findings: hemoglobin (Hb), 110 g/L (120–140 g/L), white blood cell (WBC), 6.74 × 10^9^/L (3.5–9.5 × 10^9^/L), platelet (PLT) count, 14 × 10^9^/L (125–350 × 10^9^/L); and mean platelet volume (MPV), 6.9 fl (7.4–11.0 fl). Owing to fever, thrombocytopenia and upper respiratory tract infection, the patient was diagnosed as ITP. After anti-infective therapy with dexamethasone sodium phosphate and γ-globulin, his PLT count rose to 164 × 10^9^/L and respiratory tract infection was under control. The patient was then discharged from the hospital. Two months later, his PLT count decreased to 42 × 10^9^/L, and he experienced recurrent infections, intermittent bleeding, and frequent eczema again. Blood examination showed leukocytosis of 22.82 × 10^9^/L with hematophagocytes in the bone marrow aspirate. The liver function test indicated the following findings: alanine transaminase (ALT), 113.5 U/L (9.0–50.0 U/L), aspartate aminotransferase (AST), 2 U/L (15.0–40.0 U/L), gamma glutamyl transferase (γ-GT), 137 U/L (10.0–60.0 U/L), triglycerides (TG), 2.67 mmol/L (0.56–1.70 mmol/L); cholesterol (CHO), 6.16 mmol/L (3.11–5.20 mmol/L). The results of other laboratory examinations were presented in Table 2.

Patient 2, a younger male cousin of patient 1, presented with the same clinical symptoms at 3 months of age. The laboratory test showed the following results: WBC count, 11.5 × 10^9^/L, Hb, 103 g/L and PLT count, 6 × 10^9^/L. He suffered from thrombocytopenia, intermittent bleeding, and umbilical hemorrhage and was also diagnosed with ITP. The PLT count reached 94 × 10^9^/L after treatment with γ-globulin, dexamethasone sodium phosphate and platelet transfusions. One month later, he was admitted to the hospital because of cough, expiratory dyspnea, diarrhea, and pulmonary hemorrhage. His WBC count was 16.1 × 10^9^/L and PLT count was 44 × 10^9^/L. He experienced frequent infections and died due to a fungal infection at three years of age.

Figure 1 shows the pedigree of the patients’ families. Their family history revealed that their mothers’ brother had presented with thrombocytopenia and died of infections at 3 years of age and that their mothers’ uncle was thrombocytopenic before death. No abnormalities were observed in the other family members. Therefore, the proband and his younger cousin were suspected to have WAS. On the basis of the medical and family history, they were assigned scores of 4 and 5, respectively, using a previously described scoring system [10, 14].

### Patient samples

Informed consent was obtained from the patients’ parents, legal guardians and family members before enrollment in the study. This study was approved by the Research Ethics Committee of the Second Hospital of Hebei Medical University. Ethylenediaminetetraacetic acid (EDTA)-anticoagulated venous blood samples (2 ml) were extracted from patients, their parents and carriers for mutation analysis. Heparinized venous blood samples (5 ml) were obtained from patients and carriers in their families for WASp detection.

### Flow cytometry and western blot analysis

PBMCs were isolated from the blood samples. Intracellular staining with an anti-WASp monoclonal antibody (mAb) was performed as described previously [36]. Cells were incubated with 0.25 mg/ml purified mouse anti-human WASp mAb (BD pharmingen, Franklin Lakes, NJ, USA) or 0.5 mg/ml isotype-matched control mouse IgG2a mAb (BioLegend, San Diego, CA, USA) and reacted with 1:100 diluted fluorescein isothiocyanate (FITC)-conjugated goat anti-mouse IgG2a (Affinity Biosciences, Jiangsu, China). The samples were analyzed on a BD FACSCanto II (Becton Dickinson, Franklin, Lakes, NJ, USA), using FlowJo V10 software (Becton Dickinson).

Western blotting of WASp and actin in PBMCs was performed using anti-WAS mAb (1:500; BD Pharmingen, Franklin Lakes, NJ, USA) and anti-β-actin control antibodies (1:3000; Servicebio, Wuhan, China), as described previously [37].

### Mutation analysis

Total genomic DNA was extracted from PBMCs and amplified using PCR [38]. The PCR products were sequenced by the next-generation sequencing to screen for inherited platelet disorders and immunodeficiency diseases [39]. All PCR products were sequenced using NovaSeq 6000 Genetic Analyzer (Illumina, San Diego, CA, USA). Sanger sequencing was performed on the other family members for familial segregation [40]. The forward primer (5′-CTGTCATGAGGCAGGAAGGAC-3′) and reverse primer (5′-CATCTGGATGAGTCTTTGGTTCTG-3′) were designed using Primer Premier 5. Similarity analysis was performed using the NCBI BLAST program (http://www.ncbi.nlm.nih.gov/BLAST/) to identify novel mutations. Sequences were aligned with *WAS* (NCBI reference sequence: NG_007877.1 and NM_000377.3) coding sequences.

### Construction of mutant and wild-type plasmids and cell preparation

We constructed the expressing plasmid pET-01 containing the part-length gDNA of *WAS*, which included exons 1, 2, and 3 and introns 1 and 2 as the WT. The mutation in pET-01 was constructed by site-direct mutagenesis to generate an exon mutant group and the intron mutation in pET-01 was designed to generate an intron mutant group. Both mutations involved in pET-01 were constructed to generate a combined mutant group to verify the pathogenicity of compound mutations.

Recombinant mutant plasmid was constructed with the Transformer mutagenesis kit (Clonetech, Palo Alto, CA, USA). The sequence between the two unique restriction sites, *XhoI* and *BamHI*, of each construct was verified by sequencing. Empty Vector control and recombinant WAS containing the candidate mutation in the four groups were transiently expressed in COS-7 cells (SV40 transformed African green monkey kidney cells, ATCC CRL 1651). Transfection of eukaryotic COS-7 Cells with recombinant vector DNA was performed using the DEAE-dextran method [41].

cDNA synthesis was performed using the HiFiScript gDNA Removal cDNA Synthesis Kit (Cowin Biotech, Jiangsu, China). RT-qPCR was performed using CFX connect (Bio-Rad). PCR was performed with the following parameters: 1 cycle of 15 min at 95 °C; 40 cycles of 10 s at 95 °C, 30 s at 58 °C and 30 s at 72 °C. Ten mircoliters of PCR product were mixed with the sample buffer including gel dye markers and analyzed on an agarose gel.

### Detection of the expression in mutant and wild-type recombinant plasmids in COS-7 cells

PCR products (20 μl of each PCR products including the amplified exons identified on the agarose gel) were digested with the restriction endonucleases *XhoI* and *BamHI*. Restricted DNA fragments were cloned into vectors. The inserted exons were amplified using the PCR primers 5′-CTCCAGGACCACGAGAACC-3′ and 5′-CCGTAAAGGCGGATGAAGTA-3′. The PCR products from the five groups were subjected to polyacrylamide gel electrophoresis (three replicates for each group). The cDNA products in the four groups were recovered and directly sequenced. The results were analyzed using Mygenostics software, and mutations were identified compared with the genomic gene of *WAS* (NG-007877.1) in GenBank's human genome database.

## Data Availability

Not applicable.

## Abbreviations

WAS: Wiskott–Aldrich syndrome
WASp: Wiskott–Aldrich syndrome protein
HSCT: Hematopoietic stem cell transplantation
ITP: Immune thrombocytopenia
PBMC: Peripheral blood mononuclear cell
PCR: Polymerase chain reaction
Hb: Hemoglobin
PLT: Platelet
WBC: White blood cell
MPV: Mean platelet volume
ALT: Alanine transaminase
AST: Aspartate aminotransferase
γ-GT: Gamma glutamyl transferase
TG: Triglycerides
CHO: Cholesterol
EVH1/WH1: Ena/VASP homology 1
BR: Basic region
WIP: WAS-interacting protein
WT: Wild-type
XLT: X-linked thrombocytopenia
XLN: X-linked neutropenia
HGMD: Human Gene Mutation Database
NGS: Next-generation sequencing
IPD: Inherited platelet disorders
EDTA: Ethylenediaminetetraacetic acid
